# Logics of Discovery II: Lessons from Poetry—Parataxis as a Method That Can Complement the Narrative Compulsion in Vogue in Contemporary Mental Health Care

**DOI:** 10.3390/brainsci13101368

**Published:** 2023-09-25

**Authors:** Giovanni Stanghellini

**Affiliations:** 1 Department of Health Sciences, University of Florence, 50139 Florence, Italy; giostan@libero.it; Tel.: +39-347-379-0707; 2 Centro de Estudios de Fenomenologia y Psiquiatrías, Diego Portales University, Santiago 8370068, Chile

**Keywords:** analogical thinking, Hölderlin, loss of associations, narrative compulsion, parataxis, poetry, psychotherapy

## Abstract

This paper highlights the limitations of narrative logic in mental health care, and in particular of “narrative vigilance”—the tendency to watch over experience via narrativisation, and to tether the concrete particulars of experience to the hypothetical structure of a narrative signification. Narrative logic is grounded in hypotaxis—the syntactic structuring whereby a discourse is characterised by different levels of subordination using linking words that connect, especially in terms of temporal and explanatory consequentiality. I offer an alternative approach based on parataxis—the practice of placing phrases or parts of speech next to each other without subordinating conjunctions. Sentences are juxtaposed without a clear connection; the contrast may generate novel and unexpected combinations between these dissimilar fragments. After distinguishing between parataxis and psychopathological phenomena like disturbances of association, I take inspiration from the work and life of a poet, Johann Christian Friedrich Hölderlin (1770–1843), considered among the greatest. He suffered for half his life from a severe form of mental illness that would perhaps, today, be diagnosed as schizophrenia. In the poems written during his illness, hypotaxis and narrative vigilance seem to blur, and parataxis takes centre stage. The fading of narrative structure in no way coincides with the absence of meaningfulness. Rather, meaningfulness is left to parataxis itself, that is, to the recombining power of words, sentences, and images. Parataxis itself can provide meaningfulness or, at least, provide the soil in which it can germinate. The void of narration opens the door for the fullness of “emergent” connections. In the final part of the paper, with the help of Freud’s ideas on the relationship between “analysis” and “synthesis” in psychoanalytic treatment, some implications are derived about the relevance of parataxis to the logics of discovery in psychotherapeutic care, especially that of persons with severe mental conditions.

## 1. Introduction

*Madness is to me like the unfortunate brother of poetry, it is outcast in life* [1] (p. 128).

Is it not a paradox to seek, and perhaps find, resources for the treatment of mental illness in the work of those who have been its victims, no matter how illustrious? 

This paper is a critical review of the heuristic force of narrative logic that predominates in the psychotherapeutic treatment of mental disorders. Attempting to connect the individual episodes of a person’s experience in a hypothetical narrative may result in a neglecting of the variety of content, the expressive power, and the intrinsic meaning of the episodes themselves in that person’s life. The method that I adopt to develop this hypothesis consists of analysing some salient works of a poet, Johann Christian Friedrich Hölderlin, who would probably, today, be diagnosed as schizophrenic, and resorting to a non-systematic review and analysis of philosophers and literary critics who have dealt with the ‘Hölderlin Case’ and, in particular, with the syntax—how words and morphemes combine to form larger units, such as phrases and sentences—of his writings.

The most interesting result of this review was the identification of the syntactic structure of the later works of this poet who many believe, in the second half of his life, suffered from a severe form of mental pathology. The authoritative readers of Hölderlin’s work show that what characterises his late work is *parataxis*, the practice of placing phrases or parts of speech next to each other without subordinating conjunctions. Sentences are juxtaposed without a clear connection. This contributes to Hölderlin’s work reaching unique and unprecedented evocative poetic heights and surprising perspectives of meaning. I will not go into the controversial relationship between language and thought, and between thought and perception, assuming that Hölderlin’s language reflects his way of thinking and perceiving reality and is not merely a rhetorical device. Of course, all of us (and not just people with psychopathological or neurological conditions) sometimes experience difficulty in putting our thoughts and perceptions into words, yet who but a poet would have the capacity to translate, with precision, their experiences and their syntactic structure into words and sentences?

The finding of the predominance of parataxis in Hölderlin’s late poetic works has, on the one hand, a psychopathological relevance, helping to illuminate the ‘syntax’ of the speech of a person probably suffering from a severe form of psychosis. On the other hand, it offers food for thought for clinical practice, on how to collect and organise clinical anamnesis, and for psychotherapy in particular. Building and extending on this idea, I offer an alternative approach to the clinical and therapeutic interview, based not on the connection of episodes according to a ‘narrativistic’ logic but, rather, articulated on the side-by-side juxtaposition of these episodes. Adopting an ‘emergentist’ perspective as the referential framework of my study, this ‘logic of discovery’ may, in some cases, be preferable to using the ‘narrativist’ one, as it can generate new and unexpected combinations between such fragments of experience, as harbingers of new architectures of meaning. 

In an earlier paper [2], I started developing this argument, drawing on the work of Aby Warburg, who was hospitalised for years in Ludwig Binswanger’s clinic for a form of mental insanity documented by this master of “good psychiatry” and his correspondence with his brilliant patient [3]. Warburg was affected by a psychopathological condition (which probably would be diagnosed today as “bipolar disorder”) marked by a tormenting overexcitability of the sensorium and, in particular, of the sense of smell, punctuated by furious anger, agitation, and manic excitement but, above all, by an ideation as exalted as it was fragmented, as if his self and the world were reflected in each other like a mirror shattered into a thousand pieces. This excerpt (in fact, a footnote) from a letter written during his hospitalisation on 16 July 1921 to the Directors of the Bellevue Clinic may be helpful to clarify Warburg’s psychopathological condition, and why it matters here.

“My mental illness consists in that *I lose the ability to connect* things in their simple causal relations and what is reflected in the spiritual as well as in the real so for example I can only eat simple, perspicuous food for this reason”

[3] (pp. 151–152, emphasis added).

For some reason that escapes us, Warburg’s destiny was precisely that of being able to connect “things”—in his case, works of art—far beyond the limits marked by “simple causal relations”, and finding connections that none of his fellow art historians had ever thought of.

Onto that speculation, I would now like to juxtapose another, this time inspired by the work and life of a poet, Johann Christian Friedrich Hölderlin (1770–1843), considered among the greatest, and suffering for half his life and until his death from a severe form of mental illness that would perhaps, today, be diagnosed as schizophrenia. 

What these two people had in common, in their capacity as patients and thinkers, is that they sought, perhaps precisely by virtue of their own psychopathological experience, an alternative to the logic of common sense and, in particular, to the linear narration of history and the orderly, coherent chronological organisation of events and experiences.

It is thanks to these two visionaries that we can turn critically to the logic that, in the paper preceding this one, I have called “drafting arrows”—the logic that distinguishes both common sense and the most accredited practices in contemporary psychiatry and medicine (not to mention history in general, and art or literary history in particular). To sum up: the logic of drafting arrows consists of constructing diagrams that display linear causative relationships between variables connected by an arrow to other nodes. These explanatory narratives are forms of deductive reasoning which, in psychiatry, include psychodynamic (motivational) and biological (causal) diagrams. In psychodynamic narratives, a given symptom is seen as the consequence of a given mind state, usually an unconscious motivation which, typically involuntarily, produces it. It is essential for a psychodynamic narrative to connect, via an arrow, a more “surface” or “epiphenomenal” symptom to a “deeper” mind state. Explanatory biological diagrams exploit the same logic but, in place of a psychological motivation, the antecedent to be linked to a given “surface” symptom is a “deep” biological cause.

It is beyond any reasonable doubt that the logic of drafting arrows has contributed, in the field of research, to enormous progress in the knowledge of mental disorders, and identifying psychopathological pathways leading to the formation of symptoms. The question arises, however, whether it is absolutely effective in the treatment of such disorders, and this is why I am trying to explore different “logics of discovery” that may complement narrative therapeutic practices.

In the first section of this paper, I will recapitulate the ways in which narrative logic is undermined by madness, typically in the group of symptoms characterised by the loosening or loss of associative connections. The breakdown of narrative logic is, first of all, a psychopathological phenomenon.

Later, I will argue against the limitations of narrative logic and, in particular, against what literary critic Eric L. Santner [4] has called “narrative vigilance”, i.e., the almost spontaneous tendency to watch over experience via narrativisation, and the urge to tether the concrete particulars of experience to the hypothetical structure of a narrative signification. Narrative vigilance comes at a rather steep cost: the teller overwhelms the concreteness of experience with the overarching syntax of a narrative itinerary. And the reader is pulled forward by the movement of this syntax, and the contours of the particulars tend to blur.

What I would like to demonstrate is that our authors and mentors, once they have neutralised or suspended narrative vigilance, follow a different path: in search of the soil on which knowledge will germinate, they leave the beaten track of narrative logic, do not care about “if–then” narrative connections, and sail towards other forms of “deeper” connections between mundane entities and events. The search for these forms of “darker and deeper unity”, to use Baudelaire’s words in his poem Correspondences [5], draws not infrequently on the sphere of the irrational, unscientific, unprovable, and unfalsifiable, be it mystical or metaphysical. I will show some examples, drawn from the literature and from the clinic.

However, in this paper, I would like to navigate an intermediate zone between mysticism and psychopathology, aware that this is a narrow strip, at whose edges the waters necessarily mingle. That is, I would like to leave aside questions concerning the psychopathological and the theophanic nature of these experiences and limit myself to considering the methodological aspects that can emerge from the analyses of poems and poetic theory, which can complement the narrative practices today that are in vogue in psychotherapy. 

## 2. The Dark Side: Loss of Logical Connections in Thought and Perception as Psychopathological Symptoms

Let us start by acknowledging that a whole psychopathological tradition identifies the loss of logical connections between thoughts as the core of madness. Madness is the decomposition of thought. Disturbances of associations and of thought are present in several psychopathological disorders (including, e.g., mania, melancholia, and organic psychoses) although, in each of these psychopathological conditions, they show different and specific characteristics. Eugen Bleuler describes the way in which this phenomenon is presented in people with schizophrenia in his Handbook of Psychiatry [6], in the section entitled “Schizophrenic (Dreamlike) Disturbances of Association (Zerfahrenheit of Kraepelin)”.

“Not at all infrequently new ideas crop up which have no connection of any kind with what has gone before, sometimes the patient states that they “flashed” through his mind but at other times he does not recognise anything abnormal about them. If the last-named mechanism repeatedly recurs, the mental stream becomes “distorted” and finally coherence disappears altogether. The individual thoughts then have no connection with one another from the point of view of the observer, and in most cases also from that of the patient. Indeed, it not infrequently happens that the patient never produces any coherent thought, as the concepts are piled together without any logical connection. The separation of associations from experience naturally facilitates dereistic thinking in the highest degree, which is actually based on the very fact that natural connections are ignored”.

(pp. 78–79, my italics).

### Bleuler Also Provides the Following Example from a Letter by a Person with Schizophrenia

“At the time of the New-Moon Venus stands in the August heavens of Egypt and with its rays of light illuminates the harbors of commerce, Suez, Cairo, and Alexandria. In this historically famous city of the Kalifs, there is situated in the museum of Assyrian monuments from Macedonia. There plantain flourishes next to maize columns, oats, clover, and barley, also bananas, figs, lemons, oranges, and olives. Olive-oil is an Arabian liqueur-sauce, with which Afghans, Moors, and Moslemites carry on the breeding of ostriches. The Indian plantain is the whiskey of the Parsee and of the Arab. The Parsee or the Caucasian possesses exactly as much influence over his elephant as the Moor has over his dromedary. The camel is the sport of the Jew and the Indian. In India, barley, rice, and sugar-cane, that is, artichoke, flourish luxuriantly. The Brahmins live in castes on Beluchistan. The Circassian inhabit Manchuria of China. China is the Eldorado of Pawnees”.

From the reader’s (or listener’s) point of view, the individual thoughts of the schizophrenic person are not connected to each other, they are “unzipped” (*zerfahren*), “piled together”, or merely juxtaposed to each other. “Logical” and “natural” connections are ignored, and this may facilitate “dereistic” thinking and, finally, delusions. In the face of this evidence, clinicians oscillate between two positions: either they renounce making sense of this “word salad”, limiting themselves to noting its inescapable presence as an indisputable sign of mental insanity; or they scramble to find a meaning inherent in it, a logical order in this illogical disorder, which gives or restores meaning and comprehensibility to it, but mostly according to the logical order that prevails in ordinary human affairs.

Not only do the logical connections between thoughts seems to be affected, but a breakdown in perception is quintessential, too, especially in schizophrenia. Klaus Conrad’s [7] account of the beginning of schizophrenia demonstrates the centrality of the *breakdown of Gestalt perception*, that is, a dissolution of the commonsense way to experience space. Gestalt perception includes the cohesion between the various details of a scene, and the distinction between the figure(s) in the foreground and the background. In *trema*, the uncanny atmosphere which characterises the beginning of schizophrenia, there is a disruption fundamentally altering the patient’s experience of the world, which could best be explained as a breakdown of Gestalt perception [8].

A fundamental feature of the loss of connection in the act of perception is *itemisation* [9]: the surrounding world is fragmented into a series of unrelated details. Patients describe their perceptions (especially visual ones) as “a collection of photos”, a “fragmented scene”, “things stand one next to the other”, or “seeing things like reading one line after the other without grasping the whole meaning”. The surrounding world loses its character of a meaningful ensemble, and the patient observes the appearance of fragmented details unrelated to each other and to themself. They see the trees but not the whole forest, so to speak. All this is accompanied by feelings of disconcert, perplexity, and fear but also, on the part of some patients, attraction and interest, as if they were spellbound by what is going on.

In the light of this, we may suppose that what, for the observer, is an incoherent train of thoughts may, in fact, simply reflect an incoherent perception. But we may also suppose that a person who is not able to logically connect what they experience will perceive fragments of a scene rather than a scene in its coherent entirety. The first hypothesis corresponds to a bottom-up, the second to a top-down understanding/explanation of the breakdown of associations.

It can be helpful to remember that the experience of itemisation includes three subtypes of “strange” perceptual experiences [10]:

(1) *Object fragmentation*—single objects are seen as being composed of separated individual parts, not as a whole. This can involve things or people and may include the decomposition or splitting-apart of objects not just into component parts (e.g., a chair into legs, a cushion, etc.), but also whole objects or object-parts into particles.

“I have to put things together in my head. If I look at my watch I see the watch, watchstrap, face, hands and so on, then I have got to put them together to get it into one piece” [11]. “For I saw the individual features of her face, separated from each other: the teeth, then the nose, then the cheeks, the one eye and the other. Perhaps it was this independence of each part that inspired such fear and prevented my recognizing her even though I knew who she was”. [12] (p. 37).

(2) *The break-up of a scene*—a scene or landscape is perceived as being made of individual things that appear somehow separated or disconnected from their context; the scene or surrounding world loses its cohesive unity. This may be experienced as a literal experience of the isolation of objects from one another, or as a general feeling that objects are no longer related to one another.

“Everything I see is split up. It’s like a photograph that’s torn in bits and put together again. If somebody moves or speaks, everything I see disappears quickly and I have to put it together again” [11].

(3) *The captivation of attention by isolated details*—certain details of an object or scene seem to stand out for no real reason, drawing the subject’s attention. They may include specific features (such as particular colours, lines, shapes, or textures), details, or objects that would not normally be salient or attract attention.

“Colours meant a lot to me. Stood out. No I don’t mean that there was a change in meaning, but it was just that they stood out. Especially traffic lights. I couldn’t cross the road because of this” [13].

## 3. The Breakdown of Perception and the Miasma of Unreality

In Peter Handke’s [14] novel *The goalies’ anxiety at the penalty kick*, a short novel telling about construction worker Joseph Bloch who, all of a sudden, thinks that he was fired, we can find a detailed description of the loss of connections between perceptions and the increasing sense of unreality characterising this experience. Handke’s sometimes-fractured prose conveys the atmosphere of a disintegrating world. In the space Bloch moves through, everything looks staged. Most of the things that fill up this stage-like scenario look fake, as though they were “carnival articles”; most of the events that take place look to him like “reciprocal simulations”. Bloch’s experience, which reminds the feeling of a spectator walking through a movie in which one is at the centre, is the central thread of a blockbuster film, *The Truman show*. The following is the description of the moment in which Bloch’s experience goes through an interval of respite, in which he recovers his sense of reality:

“Everything had gone well for a while after that; the lip movements of the people he talked to coincided with what he heard them say; the houses were not just facades; heavy sacks of flour were being dragged from the loading ramp of the dairy into the storage room; when somebody shouted something far down the street, it sounded as though it actually came from down there. The people walking past on the sidewalk across the street did not appear to have been paid to walk past in the background; the man with the adhesive tape under his eye had a genuine scab; and the rain seemed to fall not just in the background of the picture but everywhere”

[14] (p. 84).

Random moments of time follow one another. Sights, sounds, thoughts, and feelings do not go together. No organising principle takes successive moments in time and puts them together in a coherent way from which sense can be made. And it is all taking place in slow motion. The “miasma of unreality”, to use Louis Sass’ [15] eloquent expression, pervades Bloch’s experience: a disordered experience of time (lip movements are not synchronic with the sounds of voices), of things’ materiality (houses look like mere facades), of others (people look like crowd artists), and of space (two-dimensional rain falls) are all features of his world.

At a particularly critical moment, when Bloch seeks refuge in a room in a cheap boarding house, the perceptual fragmentation reaches its climax:

“[h]e sat down on the bed: just now that chair had been to his right, and now it was to his left. Was the picture reversed? He looked at it from left to right, then from right to left. He repeated the look from left to right; this look seemed to him like reading. He saw a “wardrobe”, “then” “a” “wastebasket”, “then” “a” “drape””

[14].

Quintessential to this pervasive feeling of fakeness and unreality is the breakdown of Gestalt space. Take, for example, this other episode in Bloch’s odyssey:

“The bus was already there, but still shut; the drivers were together at some distance and talked. Bloch sat down on a bench; the sun was shining; he ate a sandwich with a sausage…”

[14].

The reader asks, what is going on here? The scene, so itemised in particulars disconnected from each other, has no unitary significance; it simply makes no sense. Handke’s reconstruction of Bloch’s subjective experience is full of these descriptions, where a scene is fractured into snapshots that are apparently unrelated to each other. The breakdown of Gestalt space reduces the ensemble of an episode to a list of itemised details or snapshots. Each snapshot hangs next to the other, as if they were a collection of photographs lacking depth and continuity. Itemisation entails the feeling of unreality, it is accompanied by a feeling of disconnectedness from oneself and, last but not least, as meaningfulness requires the unification of details, the whole scene appears insignificant.

A final example of loss of association as a psychopathological phenomenon can be taken from Elyn Saks, a philosopher and expert through experience, encapsulating her own experience of the loosening of associations:

“There is no longer a sturdy vantage point from which to look out, take things in, assess what’s happening. No core holds things together, providing the lens through which to see the world, to make judgement and comprehend risk”

[16].

## 4. The Bright Side to the Loss of Associative Connections: Parataxis

So far, we have explored the loss of association as a psychopathological symptom, with special reference to schizophrenia. The loss of associative connections, both in thought (and related to this in speech) and in perception are, in this framework, considered abnormalities with a pathological significance.

In this section, we will see the loss of association from a different perspective: as a rhetoric device at work in literature, a particular style designed to produce a shock in the reader, or to reproduce the perception of a fragmented reality, or even a syntactic necessity to adapt language to narrate the fragmentation of *Gestalt* space.

Philosopher Giorgio Agamben [17] highlights that the core characteristic in Hölderlin’s poetry—this was also the case in his personal relationships [18,19] during the second half of his life (1806–)—was *parataxis*. 

In rhetoric, parataxis (from Greek: παράταξις, “act of placing side by side”; from παρα, para “beside” + τάξις, táxis “arrangement”) refers to the practice of placing phrases or parts of speech next to each other *without subordinating conjunctions*. Parataxis, in writing or speaking, favours short, simple sentences. It contrasts with hypotaxis (from Ancient Greek: ὐπό, hypò, ‘under’ and τάξις, táxis, ‘arrangement’), the syntactic structuring whereby the period is characterised by different levels of subordination. Parataxis typically includes no subordinating conjunctions, i.e., linking words such as “while”, “that”, “until”, and so on, but may include coordinating conjunctions. The difference between coordinating and subordinating conjunctions is a matter of dependence; coordinating conjunctions can join two or more independent ideas, whereas subordinating conjunctions show that one part is reliant on another for meaning. 

It is clear that what linguists call ‘parataxis’ is not far removed from what psychopathologists call ‘loss of associations’. If there is not a complete shared identity between these two phenomena, it should be noted at least that there is a close similarity. Some might argue that the main difference lies in the fact that, while parataxis is voluntarily sought, especially in the sphere of literature, the loss of associations is seen as a phenomenon that occurs against the person’s will.

Yet, the difference between loss of association and parataxis can be of a different kind: what in a context marked by common-sense logic might be considered a psychopathological symptom, in a context more sensitive to the magnetism that a word can exert on another word, or an image on another image, can be considered a poetic creation. The distinction between the loss of associations as a psychopathological symptom and parataxis as a stylistic choice can be the effect of a value judgement or of an aesthetical judgment.

Indeed, in literature in general, and especially in poetry, parataxis is also a *style* that consists of placing two starkly dissimilar images side by side. Two images are juxtaposed without a clear connection. The contrast may force readers to make connections between these dissimilar fragments. Some artistic forms, such as surrealistic poetry and painting, make use of this technique so as to provide a turn and a surprise for the reader or the observer. *Life at a Mid-Point* [20] is a poem written by Hölderlin exactly at the midpoint of his life, i.e., during the period in which the signs of his incipient madness became apparent. This poem is considered by literary critics an outstanding example of parataxis:

“With yellow pears hangs –
And full of wild roses –
Land hangs into the lake,
O lovely swans,
And drunk with kisses
You dip your heads
Into the sacred-sober water.
Alas, where shall I find,
When winter comes, flowers, and where
The sunlight,
And the shadows of Earth?
The walls stand
Speechless and cold; in the wind
Weathervanes clatter”
(Translation modified).

Here, the narrative vigilance seems to blur. Both formally and in terms of its content, this poem, which Hölderlin himself counts among the *Nachtgesänge*, “songs of the night”, expresses a kind of resistance to, or loss of, narrative vigilance. The form that such resistance takes in this poem is a disruption of the dialectic structure which had characterised Hölderlin’s earlier poetry. It begins with a colourful, bright, warm, paradisiac scenario and, all of a sudden, it turns it into a cold, dark, and lifeless landscape. Here, the poem abruptly stops. No overcoming, no synthesis between the scene described in the first part and the second. No redemption of the negative that follows the positive, of the death that takes the place of life.

Let us have a closer look at the “dialectic structure” that characterises much of Hölderlin’s poetry before the “turn” which seems to take place in the middle of his life. It is a ternary structure composed of a strophe followed by the antistrophe and epode. The ternary structure is also characteristic, in logic, of syllogism, a form of reasoning in which a conclusion is drawn from two given or assumed propositions or premises. Even more significantly in this context, in philosophy, a ternary structure can be found in Hegel’s dialectical method, a form of reasoning aiming to grasp the unity of the opposition between the first two determinations, or the positive result of the dissolution or transition of those determinations.

As Hölderlin himself writes about the sketch of the hymn entitled *Der Rhein*, the “law of this song” is that it is made of three parts: the first two parts are “opposite with respect to form in terms of progress and regress” and “the last one composes the whole in a universal metaphor” [21]. The dialectics of the “narrative of redemption” is imaged in Hölderlin’s vision of a secret principle migrating in cycles of plenitude and absence, from Asia to Greece and, finally, to Hesperia (the “country of the evening” designates Germany as the direction to be taken by Greek civilisation) [4]. The antitheses finally reach a conciliation. For “this” Hölderlin, time does not consume human events, but is the creator of new possibilities. Hölderlin’s meditation on history seems to have resolved itself into a sacred conception of history [22], which, according to a triadic structure, proceeds from birth and, through passion and death, arrives at resurrection.

In *Life at Mid-Point*, this triadic dialectical structure is not present. With it, the Idealist “narrative of redemption” [23] also leaves the scene. There is no development that leads from the original harmony between mortals and Celestials, through the obscuration of the Divine and the labyrinth of anguish, to a statement or an image that can recover the undermined balance by reaching a conciliatory synthesis and a kind of redemption—the metaphysical fervor which informs many of Hölderlin’s earlier poems. 

It should be noted that sharply dividing Hölderlin’s poetic production into two opposite periods is, at least, imprecise. As an example of the vestiges of the narrative of redemption in the last part of his life, we can take this short poem dedicated to Herr Zimmer, the carpenter who housed him as a boarder in his own house and took care of him:

“The lines of life are various,
Like roads, and the borders of mountains.
What we are here, a god can complete there,
With harmonies, undying reward, and peace”.

Yet, at a certain point of his life, the narrative of redemption is replaced by parataxis, which “becomes the principle which informs not only the patterning of images within the strophes, but the side-by-sideness, the harsh juxtaposition of the two strophes, which make up the poem as well” [24]. Paratactic composition will juxtapose (place side by side) what the dialectic would otherwise synthesise and absorb into one sweeping hypotactic movement. The poet distinguishes two forms of infinitude, one “empty” and “deadly”, the other full, vital, “total and unitary”. The former consists of infinite isolated elements, while, in the latter, the elements are “more infinitely connected”—but *not* according to a logical arrangement [17].

Redemption is not looked for in a logical arrangement. This renunciation to a narrative (dialectic) synthesis—and this what matters most here—by no means implies a surrendering to scepticism, despair, or nihilism. The infinite space left by the absence of narrative redemption in no way coincides with the absence of any hope of redemption.

Rather, *redemption is left to the parataxis itself*, that is, to the recombining power of words and images. Parataxis itself can provide meaningfulness or, at least, provide the soil in which it can germinate. The void of narration opens the door to the fullness of “emergent” (as we might dare to say, using this adjective anachronistically) connections. Recombination occurs in nature as an essential part of the generation of life. The purpose of art, according to Hölderlin, is to pave the way to *unendlicher Zusammenhang* (infinite connections), to “make the infinite present”, something that is only possible if the work is a “living form”, a “still point” from which harmonic alternations and resonant oppositions radiate.

Parataxis itself helps to plough this soil and make it fertile for a possibly infinite variety of combinations of meanings. *Harte Fügung* (literally: “hard coincidence”), in Norbert von Hellingrath’s words, makes it possible for individual words themselves to establish a relational unity, rather than unity being imposed by a legislating subject [23].

## 5. Another Kind of Dialectics: Synthesis Spontaneously Following Analysis in Psychopathology and Psychotherapy

As we have seen earlier in this paper, in itemisation the surrounding world is fragmented into a series of unrelated snapshots, losing its character of a meaningful ensemble, and the patient is spellbound by the appearance of fragmented details unrelated to each other and to himself. So to speak, he cannot see the forest for the trees.

This phenomenon, typically at a later stage, may turn into its opposite: all details *hang together* and lead to an experience of sudden revelation. Klaus Conrad [7] reports that the “cloud” of the fragmented details suddenly hangs together, and the hidden meaning that hides within all objects comes to a manifestation; Wetzel [25] shows that the fragmentary intimations that twinkle in the experience of itemisation can shape up into a full-blown revelation experience.

The serial killer Moosbrugger, one of the characters described by the Austrian novelist Robert Musil in his masterpiece *The Man without Qualities* [26] (who, like Handke’s Bloch would probably justify a diagnosis of schizophrenia), explains that nothing in the world can be singled out because all things hang together:

“A squirrel in these parts is called a tree kitten”, it occurred to him, “but just let somebody try to talk about a tree cat with a straight face!”. “In Hesse, on the other hand, it’s called a tree fox. But oh, how curious the psychiatrists got when they showed him a picture of a squirrel and he said: “That’s a fox, I guess, or it could be a hare, or maybe a cat or something”. Moosbrugger’s experience and conviction were that no thing could be singled out, because things hang together” (p. 259).

Is this the description of a spontaneous “synthesis” following an experience of loss of associations?

Are hang-togetherness and itemisation the two psychopathological sides of the same coin? Is hang-togetherness the extremisation of a sort of “narrative reflex”, in which the stimulus is represented by the loss of coherence, and the response is the apperception of a profound and invisible unity? If, on one hand, we can say that hang-togetherness is a psychopathological symptom, can we say the same about the “narrative reflex”, or is it a property of every human being, and a resource for psychotherapeutic care?

Sigmund Freud, in *Lines on the Advances of Psycho-Analytic Therapy* [27], illustrates the dialectics between analysis and synthesis in the process of psychotherapeutic care.

“The work by which we bring the repressed mental material into the patient’s consciousness has been called by us psycho-analysis. Why ‘analysis’—which means breaking up or separating out, and suggests an analogy with the work carried out by chemists on substances which they find in nature and bring into their laboratories? (…) This well-founded comparison of medical psycho-analytic activity with a chemical procedure might suggest a new direction for our therapy. We have analysed the patient—that is, separated his mental processes into their elementary constituents (…); what could be more natural than to expect that we should also help him to make a new and a better combination of them? (…) We have been told that after an analysis of a sick mind a synthesis of it must follow (…) The comparison with chemical analysis has its limitation: for in mental life we have to deal with trends that are under a compulsion towards unification and combination. Whenever we succeed in analysing a symptom into its elements, in freeing an instinctual impulse from one nexus, it does not remain in isolation, but immediately enters into a new one. (…) The psycho-synthesis is thus achieved during analytic treatment without our intervention, automatically and inevitably”

[27] (pp. 160–161).

After more than a century, these ideas remain powerfully challenging. Let us summarise them: (1) clinical work consists mainly of an “analysis” of the material provided by the patient, that is, separating the “elementary constituents” of mental phenomena; (2) it is not necessary to pass from this “analysis” to a “synthesis”, i.e., to actively recombine the pieces into a whole; (3) this recombination is spontaneously provided by the mind, as it is endowed with a “compulsion towards unification and combination”—something that reminds us of Santner’s “narrative compulsion”?—which is realised “without our intervention, automatically and inevitably”.

Freud’s concluding remarks about the “fundamental principle”—to carry on the analytic treatment “under privation” and in a “state of abstinence”—may remain blurry. Yet, in this half-light, we see a glow filtering through that reverberates with parataxis:

“You will observe—writes Freud—that this opens up a new field of analytic technique the working over of which will require close application and which will lead to quite definite rules of procedure. I shall not attempt to-day to introduce you to this new technique, which is still in the course of being evolved, but will content myself with enunciating a fundamental principle which will probably dominate our work in this field. It runs as follows: Analytic treatment should be carried through, as far as is possible, *under privation—in a state of abstinence*”

[27] (p. 162, my italics).

Which is the “new field of analytic technique”, “still in the course of being evolved” that Freud alludes to? Remember that we are in 1919, just at the mid-life of Psychoanalysis… Is Freud suggesting something akin to “linking dots”, and the therapeutic use of parataxis instead of hypotaxis and narrative thinking?

And, also, “privation” of what? And “abstinence” from *what*? Perhaps from the narrative compulsion? From the logic of deductions? From hypotactic reasoning? From standard syllogisms and subsequent impersonal interpretations?

With all that in place, we can turn to some concluding remarks.

## 6. Conclusions: Parataxis—How to Set the Forest on Fire without Burning the Trees

In this paper, I address the phenomenon of parataxis in psychopathology and mental health care through the analysis of Hölderlin’s poetic work and the contribution offered by philosophers and literary critics.

I define ‘parataxis’ as the bracketing of connections between the parts of a discourse, letting them float rather than be immovably grounded in pre-established narrative relations.

I argue that the absence of an obvious connection between the parts of a discourse is not necessarily, in itself, a psychopathological symptom. In some case, it may reflect the absence of connection of the perceptual experience that one intends to narrate and, in this case, the anomaly is to be found in the perceptual realm, not in the narrative or linguistic realm. In other occasions, it may represent a deliberate attempt to make the linguistic form resemble the form of perception. Or, finally, it may represent an attempt to unhinge the ordinary logic present in perception and/or language, in search of connections of another kind.

Some of Hölderlin’s poems in his late production, as well as his answers to the visitors in the tower, are surprising, disorientating, and seemingly incoherent compositions of individual aphoristic fragments and short sayings, in which dazzling images and laconic sentences are alternated. The obstinate parataxis present in Hölderlin’s late literary production may be a consequence of either the disorganisation of his perceptual experience, or an anomaly on the linguistic and narrative level or, finally, a combination of the two. However, the important anticipations we find in his poetic theory lead us to think that parataxis in Hölderlin may represent a deliberate choice [17].

The main issue that I addressed can be condensed into the question: can parataxis be seen as a *method* of discovery? My answer is that, if we see it as a method, it may shed further light on the emergentistic logic that I called “linking dots” [2], its heuristic power, and its potential in the therapeutic sphere.

The ‘logic of discovery’ offered by parataxis resonates *ante litteram* with the montage in surrealistic figurative art and literature, and with the methods of discovery at work along anti-historicist thinkers like Aby Warburg and Walter Benjamin.

The obstinate parataxis that Agamben and other readers of Hölderlin find at work in the “tower poems” also evokes Warburg’s “atlas of images” and Benjamin’s “constellations of images” [28,29]. It also brings to mind “allegory” (the allegory, from *allos* ‘other’ + *agoreuo* ‘speaking’)—allegories “speak of something else”; they express unexpected meanings by “disassembling” and “reassembling” a common-sense discourse [30]. All this brings to the fore the power of images, and not only of concepts and narratives, to represent and understand psychopathological conditions.

To a certain extent, parataxis is also related to Freud’s ideas about the need to carry on analytic treatment in a state of “abstinence”—presumably not abstinence from sex, but from ordinary interpretations and narrativistic constructions. Certainly, parataxis resonates with the procedure of free associations, as formulated by Freud in *Studies on Hysteria* [31]. With the method of free associations, the insistent search for the pathogenic element (which characterises the markedly hypotactic pre-analytic techniques) gives way to the spontaneous expression of the patient, which favours the paratactic juxtaposition of memories, images, etc. (see Table 1), and, thus, the *emergence* of unforeseen meanings via the proliferation of a network of associations.

My hypothesis is that there can be a teleology at work in parataxis. To use a metaphor, parataxis acts like a fire that burns the forest without burning the trees. Without the forest that imprisons them, the trees become free to configure new Gestalten. And this helps the observer and the clinician to open up to emergent unlimited possibilities of meanings.

Emergence occurs in the first place when an entity is observed to have properties that its parts separately do not, properties that emerge only when the parts interact on a wider whole. An emergent phenomenon can be roughly defined as a phenomenon that is irreducibly *relational*; emergent features are not reducible to any intrinsic property of any element of a whole. Emergentism seems to be a corrective that counteracts the limitations of narrative compulsion.

The absence of connection, or harsh connection (*harte Fügung*), becomes a real principle of poetic composition, surely as an attempt to overcome an ordinarily logical connection between sentences or parts of a poem. Hölderlin sought to articulate the connection between thoughts in a non-traditionally logical way—not based on premises and consequences united by becoming, purpose, and end, but held together by secondary propositions consequentially linked to the main ones. Are we allowed to think that Hölderlin was trying to overcome the logic of drafting arrows, i.e., the telling of a linear and continuous story? We can see in other poems that this is a narrative of redemption connecting experiences of plenitude followed by absence, finally making sense of the latter as the necessary gate to a new and deeper experience of plenitude.

**Figure 1:**
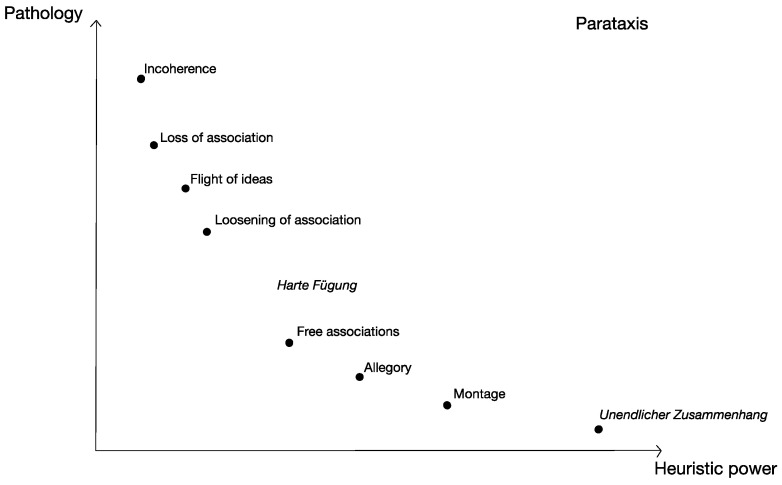
**Parataxis—its heuristic power and pathology.**

If those critics who assign a central role to parataxis in Hölderlin’s work in the period of the “tower poems” were right, then Hölderlin—or his madness, or more likely both—would have to be considered the true initiator of a *nouvelle vague* of poetry and art that, through Baudelaire, reaches all the way to Surrealism and to the present day. Indeed (and this is more relevant in our context), this is not just a new way of making poetry, but a new method to achieve knowledge. The poetic (or, at least, heuristic) effect arises from the benevolent hospitality given to chaos, to the swarming of images, forced by apparently fortuitous juxtapositions to hybridise in combinations never before experienced. The poetic effect is the effect of what chemists call “unstable compounds”—a very high affinity between discordant elements [32] (see Figure 1).

One might even think that, in the last part of his life, spent in a solitary tower over the Neckar River in Tübingen, his polemical target was *History itself*, understood as the narration of the logical and consequential succession of events. One might imagine that, in this way, he was claiming his right to *call himself out of History*, which, indeed, he did in the second half of his life. Baudelaire would have said that, among the fundamental freedoms of man, comes one that is forgotten and, that is the freedom to call oneself out:

“in the several enumerations of the rights of man which the wisdom of the 19th century renews so often and with such satisfaction, two rather important ones have been forgotten which are the right to contradict oneself and the right to leave”

[33].

Hölderlin seems to have taken this ironic and bitter statement by Baudelaire literally, anticipating it. “Calling oneself out of history” can be taken for a psychopathological symptom—*autism*. In Hölderlin’s case, it may not only be a “negative” symptom, but the outcome of a “different will”. So, this “calling oneself out” of history and narrative logic was not a mere renunciation, but an aspiration to something higher. An aspiration far removed from nihilism and relativism but, rather, a wish to grasp complexity, “the natural obscurity of things” [34], a higher form of cohesion between them, and a more infinite connection in an infinite unity.

What remains of all this in the memory of a psychiatrist, trained to use structured interviews to establish a reliable diagnosis, and prescribe medications (be they drugs or psychological techniques) that also draw from the domain of logical consequentiality?

The impression remains that there is a form of knowledge of nature, and a kind of relationship with it, that is not strictly technical, and does not exploit causal–explicative or motivational–comprehensive logic, but draws on different principles of knowledge and action, which must never stand as adversaries of technique (to which we owe much), but as its integration. I refer to *analogical thinking*, of which poetry is the master, and which may show us connections between things and events that cannot be noticed otherwise.

‘Analogy’ is an ill-famed word among men of science but, among poets, the music that accompanies its chords is quite different: “*imagination* is the most *scientific* of faculties, for it is the only one that understands *universal analogy*, or what a mystical relay calls *correspondence*”, in the words of Charles Baudelaire [35].

In the worst case, knowing the laws of analogy can help us recognise the thought structures of some of our patients. Therefore, this journey through parataxis can, at least, help us to understand something more about “logics” other than those of common sense. But what is at stake here is not just understanding a certain way of thinking, but learning how to use it for the benefit of patients. Of course, one can only agree with Goethe’s wisdom: “If one follows analogy too closely, everything coincides in the identical; if one avoids it, everything disperses into infinity” [36]. It is difficult to find the balance between the “too much” and “too little” analogies—just as we saw earlier, it is difficult to find a clear dividing line between loss of associations and parataxis. Nevertheless, it cannot be denied that analogies are widely recognised as playing an important heuristic role as aids to discovery. They can create a space for knowledge within which darkness—what, to technique-driven computational and narrative procedures, remains invisible—becomes visible:

“a great Furnace flam’d, yet from those flames
No light, but rather darkness visible”
[37].

## Figures and Tables

**Table 1:** Inventory of paratactic and hypotactic phenomena.

**PARATAXIS**	
**Incoherence**	the total breakdown of logical connections
**Loss of association**	a formal thought disorder characterised by a lack of association between different ideas, resulting in disorganised thinking
**Flight of ideas**	a typically exuberant loosening of associations
**Loosening of associations**	inconsequential (e.g., tangential) thinking
***Harte F*** ***ügung* (sensu von Hellingrath)**	a “hard coincidence” between thoughts or images
**Free associations (sensu Freud)**	the spontaneous expression of at-face-value unrelated mental contents
**Allegory (sensu Benjamin)**	“speaking of something else”, expressing a meaning by disassembling/reassembling a common-sense discourse
**Montage (sensu Benjamin/Warburg)**	a shock-inducing juxtaposition/contraposition of sentences or images which shows similarities, correspondences, contrasts, etc., that would otherwise have remained latent
***Unendlicher Zusammenhang* (sensu Holderlin)**	infinite connections between thoughts and images
**HYPOTAXIS**	
**Narrative vigilance**	the tendency to watch over experience via narrativisation, to tether the concrete particulars of experience to the hypothetical structure of a narrative signification
**Narrative compulsion**	the extremisation of narrative vigilance towards unification
**Hang-togetherness**	the sudden coalescence of a “cloud” of previously unrelated fragments with the revelation of a hidden meaning
**Systematic delusion**	a highly organised “abnormal” belief with multiple elaborations that are coherent, consistent, and logically related

## Data Availability

Not applicable.
